# Inhibition of Neutral Sphingomyelinase 2 by Novel Small Molecule Inhibitors Results in Decreased Release of Extracellular Vesicles by Vascular Smooth Muscle Cells and Attenuated Calcification

**DOI:** 10.3390/ijms24032027

**Published:** 2023-01-19

**Authors:** Angelina Pavlic, Hessel Poelman, Grzegorz Wasilewski, Kanin Wichapong, Petra Lux, Cecile Maassen, Esther Lutgens, Leon J. Schurgers, Chris P. Reutelingsperger, Gerry A. F. Nicolaes

**Affiliations:** 1 Department of Biochemistry, Cardiovascular Research Institute Maastricht (CARIM), Maastricht University, 6200 MD Maastricht, The Netherlands; 2 Amsterdam UMC, University of Amsterdam, Medical Biochemistry, Meibergdreef 9, 1105 AZ Amsterdam, The Netherlands; 3 Institute for Cardiovascular Prevention (IPEK), Ludwig-Maximilians Universität, 80539 München, Germany; 4 German Center for Cardiovascular Research (DZHK), Partner Site Munich Heart Alliance, 80539 Munich, Germany; 5 Cardiovascular Medicine, Mayo Clinic, 200 First St SW, Rochester, MN 55905, USA

**Keywords:** drug discovery, virtual ligand screening, vascular calcification, SMPD3, nSMase2, small molecules, extracellular vesicles

## Abstract

Vascular calcification (VC) is an important contributor and prognostic factor in the pathogenesis of cardiovascular diseases. VC is an active process mediated by the release of extracellular vesicles by vascular smooth muscle cells (VSMCs), and the enzyme neutral sphingomyelinase 2 (nSMase2 or SMPD3) plays a key role. Upon activation, the enzyme catalyzes the hydrolysis of sphingomyelin, thereby generating ceramide and phosphocholine. This conversion mediates the release of exosomes, a type of extracellular vesicles (EVs), which ultimately forms the nidus for VC. nSMase2 therefore represents a drug target, the inhibition of which is thought to prevent or halt VC progression. In search of novel druglike small molecule inhibitors of nSMase2, we have used virtual ligand screening to identify potential ligands. From an in-silico collection of 48,6844 small druglike molecules, we selected 996 compounds after application of an in-house multi-step procedure combining different filtering and docking procedures. Selected compounds were functionally tested in vitro; from this, we identified 52 individual hit molecules that inhibited nSMase2 activity by more than 20% at a concentration of 150 µM. Further analysis showed that five compounds presented with IC_50_s lower than 2 µM. Of these, compounds ID 5728450 and ID 4011505 decreased human primary VSMC EV release and calcification in vitro. The hit molecules identified here represent new classes of nSMase2 inhibitors that may be developed into lead molecules for the therapeutic or prophylactic treatment of VC.

## 1. Introduction

Vascular calcification (VC) is characterized by mineralization of vascular tissues and is a highly prevalent pathological process in the aging population of industrialized countries [1]. VC is a risk factor and predictor of cardiovascular morbidity and mortality [2]. Despite its relatively high prevalence (37.5% in an average population) [3] and its detrimental role in cardiovascular disease, no therapies are currently available that specifically target VC [4].

VC can occur in both the intimal and medial layer of arteries, also referred to as calcific atherosclerosis and arteriosclerosis or Mönckeberg’s sclerosis, respectively [5]. VC results from a disturbed balance between the pathways that transport and deliver calcium (Ca^2+^) intracellularly and those that prevent and minimize precipitation of calcium phosphate salts in extracellular fluids. Vascular smooth muscle cells (VSMCs) play an important role in the regulation of VC [6]: as a result of increased extracellular Ca^2+^, they secrete extracellular vesicles (EVs) [7,8,9], which have the capacity to promote VC [10]. EVs can exhibit pro- or anti-calcifying activity, depending on their quantity, composition, and cargo, which depend on their microenvironment and on the phenotype of the VSMCs from which they derive [8,11].

Of relevance to this, neutral sphingomyelinase 2 (nSMase2, or NSMASE2), also known as sphingomyelin phosphodiesterase 3 (SMPD3), is a member of the family of neutral SMases. It has been identified as a key enzyme in the biogenesis and cargo loading of exosomes, a type of EV [8,12]. nSMase2 hydrolyzes the conversion of sphingomyelin into phosphocholine and ceramide, which triggers the formation of exosomes in multivesicular bodies [13]. Pharmacological inhibition, knock-down or knock-out of nSMase2 decreases exosome release in a variety of cell types, indicating a fundamental role of nSMase2 in biogenesis and release of exosomes [14]. Inhibition of nSMase2 by fetuin-A reduced secretion of pro-calcifying EVs by VSMCs and prevented their calcification in vitro [8]. Hence, nSMase2 is a potential target for the pharmacological modulation of VC. To date, only few inhibitors (e.g., GW4869, manumycin A, spiroepoxide, PDDC, and cambinol) of nSMase2 have been reported in literature; however, they lack specificity or have high IC_50_ values [13,15,16,17,18] in the micromolar to millimolar ranges [19]. Thus, there is a need to widen the spectrum of available nSMase2 inhibitors, preferably with low IC_50_ values and with specificity for the nSMase2 isoform.

Here, we present 2 novel nSMase2 inhibitors, which are characterized by IC_50_ values in the low µM range. These inhibitors were discovered through application of a structure-based virtual screening method as applied by us in a previous study [20], combined with in vitro and ex vivo screening methods.

## 2. Results

### 2.1. The Human nSMase2 Structure Has Druggable Pockets

**Figure 1:**
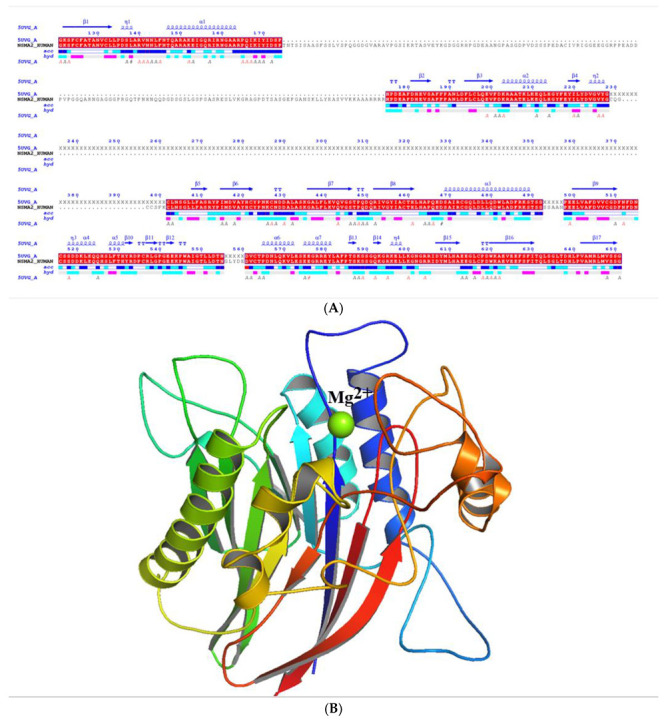
**nSMase2 structure preparation.** (**A**) Sequence alignment of the amino acid sequence from PDBId:5UVG and the sequence of hSMAse2 with EndScript2 [21], applying EMBOSS Water with the Smith-Waterman algorithm using standard parameters. Identical residues are highlighted in red. (**B**) Ribbon representation of the 3D model of human nSMase2 catalytic domain with Mg^2+^ (green sphere) in the active site and the protein colored in N-to-C mode (blue-to-red).

We generated a complete target structure by molecular modelling, based on comparison of the sequence of human nSMase2 and the amino acid sequence from PDBId:5UVG (Figure 1).

**Figure 2:**
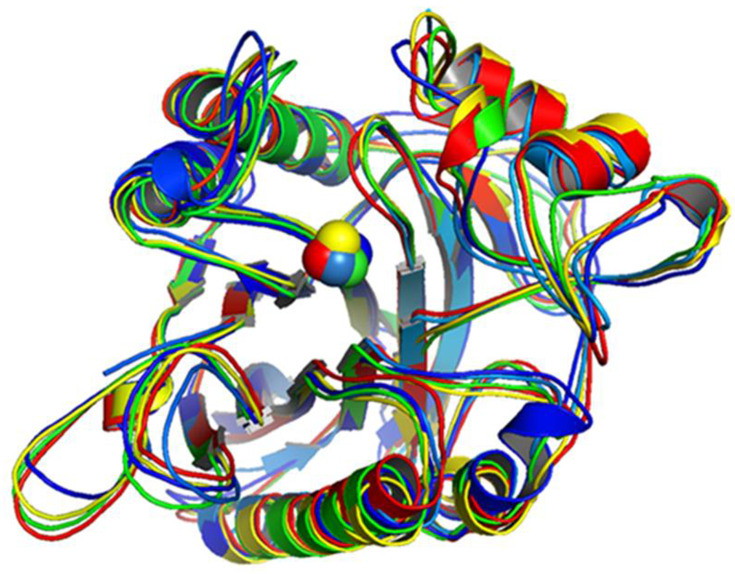
**Superposed nSMase2 structures.** Superposed crystal 5UVG and MD-derived structures after 10 ns (dark blue), 20 ns (light blue), 30 ns (neon green), 40 ns (spring green), 50 ns (light green), 60 ns (yellow), 70 ns (dark yellow), 80 ns (light orange), 90 ns (orange), 100 ns (red), 5UVG (sky blue).

To evaluate the rigidity of the nSMase2 catalytic domain structure and to inspect its druggability, we performed MD simulations. This also allowed us to derive an ensemble of diverse structures for virtual screening. After the MD run, the trajectories were visually inspected and 10 different conformations from every 10 ns pose were selected. The resulting 10 conformations were superposed, and we visually analyzed the metal ion which coordinates the binding residues of the protein (Figure 2). The overall structures were similar; however, the metal ion coordinates slightly deviated from the original position, implying limited flexibility of the amino acid side chains within the binding pocket during the simulation. Further analysis involved the binding pocket definition based on comparison of the 10 structural protein conformers derived from the MD run.

To define the presence and positions of pockets, we used DoGSiteScorer to analyze the 10 structures [22,23]. Based on the selection criteria, including pocket volume, and drug score, DoGSiteScorer identified the active site as the most suitable druggable pocket (Table 1). We then selected the seven structures with the largest pocket volume for further use in our virtual screening protocol and complemented these with the MD starting structure. The structures selected were those after 80 ns, 70 ns, 20 ns, 10 ns, 90 ns, 40 ns and 50 ns, in order of decreasing size, with the 50 ns structure having the highest drug score of 0.86 (Table 1).

### 2.2. Enzymatic Assay Revealed Five Strong nSMase2 Inhibitors

Based on the structural modelling we discovered that nSMase2 activity may be amenable to modulation by drugs. Therefore, we decided to examine the effects of the compounds selected in the virtual screening steps, which were successfully docked into the nSMase2 druggable pocket, on nSMase2 activity in vitro. Mixtures of four randomly picked compounds were prepared and tested. As a cut-off value for presence of inhibitory activity, we used an arbitrary threshold of 20% inhibition. When an inhibition of >20% was found, the mixture was deconvoluted and each of the four compounds was tested individually. We thus identified 279 individually tested compounds that possessed activity, of which 68 compounds inhibited the enzymatic assay by >20%. Since the assay used included sphingomyelinase and three other enzymes (alkaline phosphatase, choline oxidase, and peroxidase), we assessed interference with other components of the assay by testing the 68 hit compounds, while excluding nSMase2 and sphingomyelin, and adding phosphorylcholine (natural ALP substrate) instead. This resulted in the exclusion of 16 compounds, as they influenced the assay outcome even in the absence of nSMase2 and, therefore, represent false positives.

**Figure 3:**
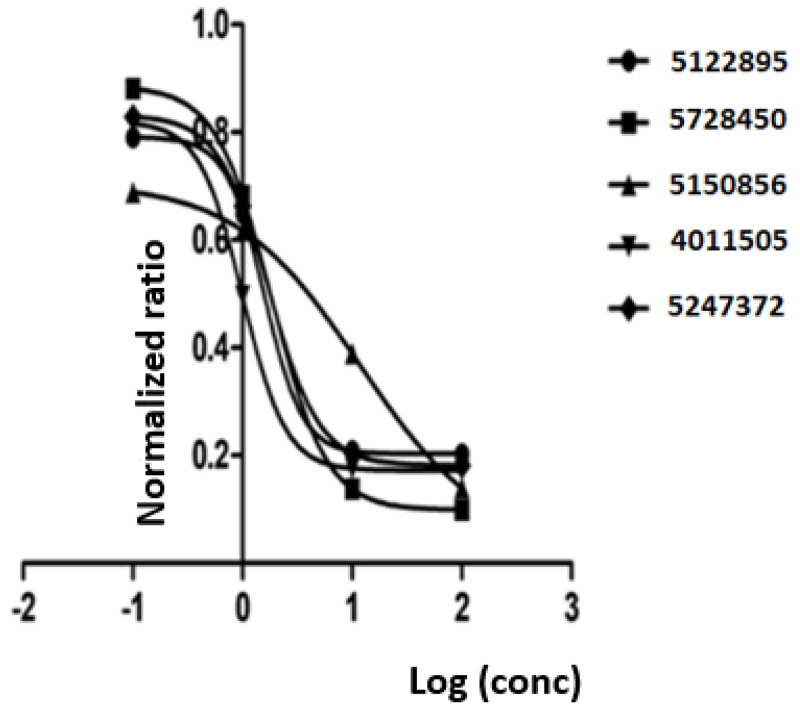
**Dose-response in enzymatic assay for five inhibitors with the lowest IC_50_ values.** Dose-response is shown as the log of concentration (in microMolar) in relation to a normalized ratio.

Thus, we identified 52 out of 996 compounds as inhibitors of nSMase2, implying a hit rate of 5.2%. Of note, the enzymatic assay also revealed 23 compounds that increased sphingomyelinase activity. While interesting, these compounds were not evaluated further. All the identified active compounds dissolved well in DMSO and were tested under identical experimental conditions as described in the Methods section. Next, the five strongest inhibitors that were assayed at a concentration of 150 µM were titrated at concentrations ranging from 0.1 µM to 100 µM, to determine their IC-50 values. Their IC-50 values ranged from 1.0 µM to 11.7 µM (Table 2, Figure 3).

### 2.3. Chemical Diversity Clustering Reveals Two Druglike Inhibitors of nSMase2

The five most active compounds, as judged by their IC_50_ values, were further analyzed for their druglikeliness with SwissADME [24]. This revealed compounds ID 5728450 and ID 4011505 as the best candidates, based on their possession of low IC_50_ values and only one violation of the general rules for drug-likeliness, in relation to their lead-like qualities (MW < 250 Da; (Figure S2A–E). Next, we applied cluster analysis to gain insight into their structure-activity relationship (SAR). To this end, all 996 compounds that had been tested in the enzymatic assay were clustered with MURCKO scaffold in Data Warrior [25], at a similarity limit of 80, analyzing activity cliffs. The cluster groups were separated by structure. We found 411 different clusters with one or more member compounds (Figure S3A). In this setup, compound ID 4011505 shared a common scaffold activity with only one other compound in its cluster (Figure S3B), differing only in the position of one methyl group. Compound ID 5728450 clustered with 12 other compounds (Figure S3C).

For this study, to obtain approximate SAR information besides the two inhibitory compounds ID 5728450 and ID 4011505, we selected an additional two inactive compounds, ID 540122 and ID 5784643, and one activator, ID 6924649, for further experiments in VSMCs (Table 3). ID 540122 is structurally similar to ID 5728450 and belongs to the same cluster, 103. ID 5784643, from cluster 154, is structurally different than the inactive compound ID5402122 in cluster 103. The activator, ID 6924649, does not share similarities with any other compound.

### 2.4. Binding Mode of Inhibitors

The five hit molecules that were characterized via the in vitro enzyme assay used (Table 2) and the selected two inactive analogues and one analogous activator were re-docked on the nSMase2 target structure with Surflex, to obtain additional insight into the mode of binding of these molecules. Surflex produced 10 poses for each compound and, after scoring, returned a top scoring pose for each of the compounds tested.

We manually compared all poses and inspected their geometries to detect common protein–ligand interaction motifs. Inspection of the docked poses revealed that there was a difference between the inhibitors 5728450, 5122895, 5274372, 5150856, and 4011505 on the one hand, the structurally similar inactive 5402122 and unrelated inactive 5784643 on the other hand, and, finally, the activator tested, 6924649.

**Figure 4:**
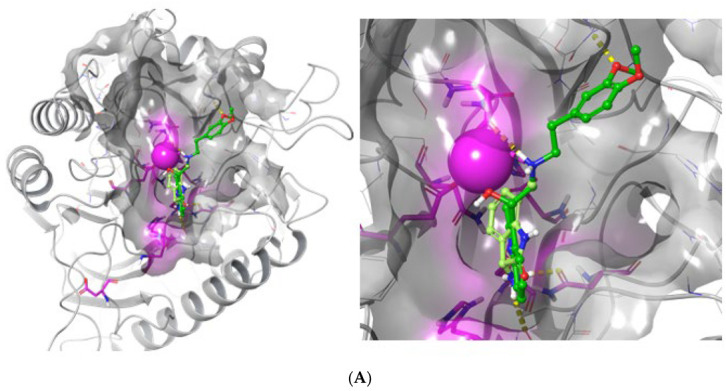

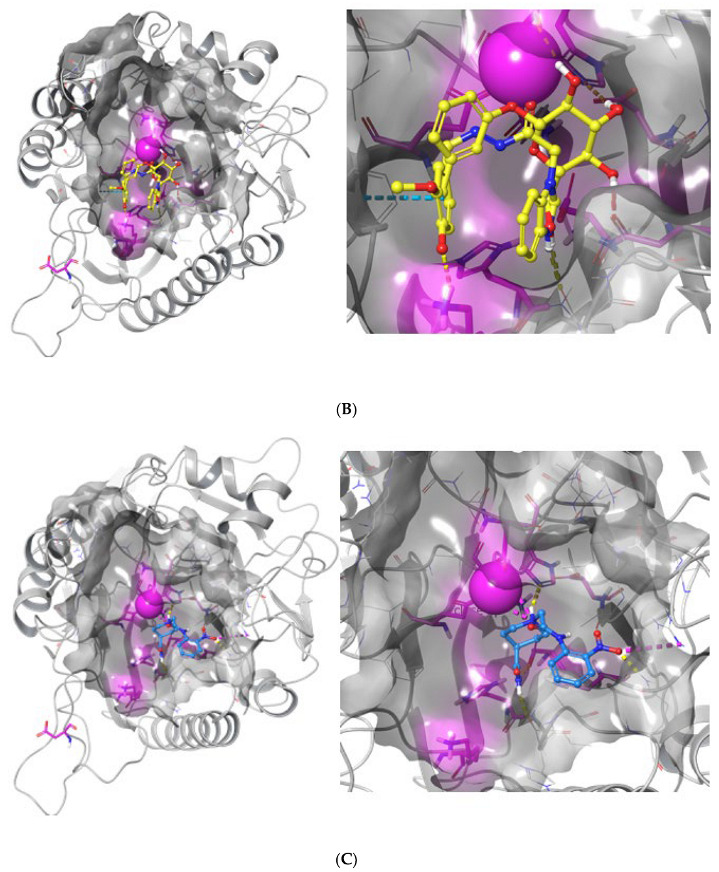
**Docking pose of compounds 4011505, 5122895, 5402122, 5784643, and 6924649.** (**A**) docking pose for the best two inhibitors, compounds 4011505 (light green) and 5122895 (green), in 3D presentation. (**B**) Docking pose for the two inactives, compounds 5402122, and 5784643 (yellow). (**C**) Docking pose for the activator 6924649, tested in blue. The catalytic Mg^2+^ ion is shown as a magenta sphere, with the position of the conserved active site residues important for catalytic activity [26] indicated as magenta shading. The so-called DK-switch [26] with residue D430 is indicated as a magenta sidechain for orientation. The surface of the active site pocket is overlaid on the ribbon structure of SMase2. The insert shows a close-up of the active site region in 4a, 4b and 4c.

Figure 4A–C below illustrates the top-ranking poses that were obtained for the two best activators (4011505 and 5274372; Figure 4A), the two inactives (5402122 and 5784643; Figure 4B) and the activator tested (6924649; Figure 4C). Individual poses are found in supplemental Figure S4A–H. Overall, the activating and non-activating compounds appeared to dock in a similar pose, yet their topologies appeared to differ. We found that all inhibiting compounds, 5728450, 5122895, 5274372, 5150856, and 4011505, showed a binding pose that positioned the compound in the zone I (cf. Figure 7); compound 5402122, which is structurally similar to compound 5728450 and compound 5784643, were predicted to bind in the zone II; and the compound 6924649, which showed a stimulatory activity, bound in the zone 3.

Interestingly, we noted that the compounds 5728450 (inhibitor) and 6924649 (activator) showed very similar binding poses; however, 6924649 appears to penetrate deeper into a local subpocket, pointing towards residue Asn512 at the bottom of this pocket.

**Figure 5:**
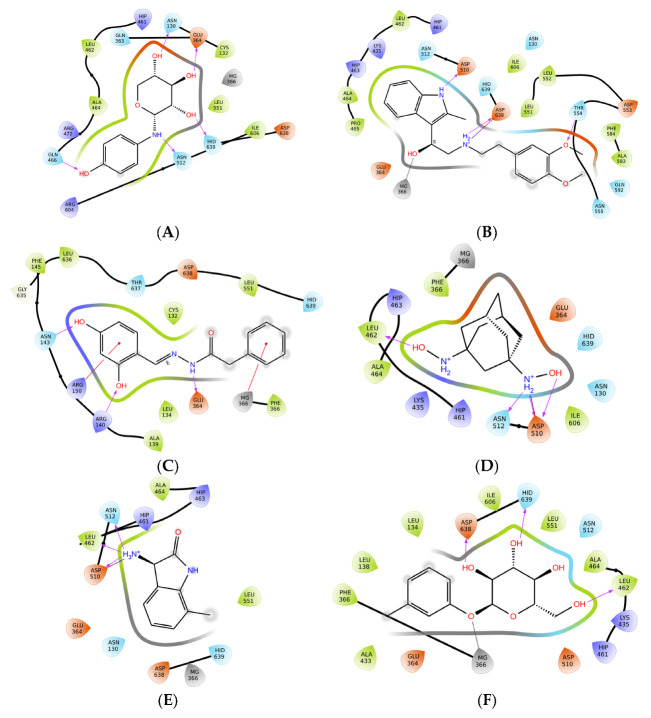

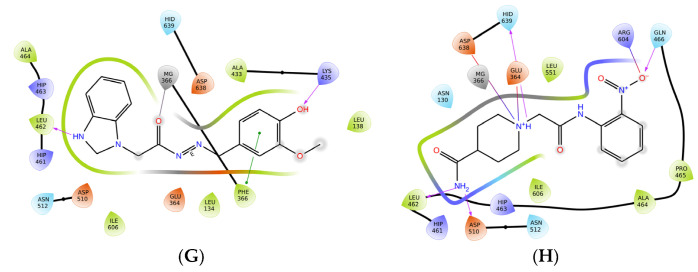
(**A**–**H**): **2D representation of docking poses after redocking.** Shown are the molecules 5728450 (A); 5122895 (B); 5247372 (C); 5150856 (D); 4011505 (E); 5402122 (F); 5784643 (G) and 6924649 (H) interacting with nSMase2 in a 2D representation with polar residues (light blue), negatively charged residues (orange), positively charged residues (dark blue), hydrophobic residues (light green), metals (grey), H-bond (purple arrow), π–π stacking (green lines), π–cation (red lines), solvent exposure (light grey cloud), displaced hydration site (X), salt bridge (blue to red line).

To allow for a more readily readable insight into individual interactions that summarizes the interaction data from the 3D poses, Figure 5A–H illustrates the 2D interaction graphs that can be extracted from the binding poses for each of the compounds. It can be observed that interaction with the active site Mg^2+^ ion in itself does not distinguish activators from non-active compounds, since both non-active compounds contact the catalytic metal ion, whereas only 2/5 of the inhibitors apparently contact the Mg^2+^. The conserved active site residues Asn130, Glu364, Asp510, Asn512, Asp638, and His639, as were presented by Airola and coworkers [26], are involved in the residues that are identified here; however, no conclusive observation can be made as to contribution of any of these residues individually.

Analyses of interactions (see Table 4 below) indicated that all inhibitors except 4011505 showed more interactions with the nSMase2 active site than the two inactives (cf. 5 vs. 4 contacts), yet the activator showed seven contacts, suggesting it to be bound more extensively than the inhibitors. The number of bonds is, however, not in itself sufficient to explain the observed activities.

### 2.5. nSMase2 Inhibitors Reduce VSMCs Calcification and EV Release

**Figure 6:**
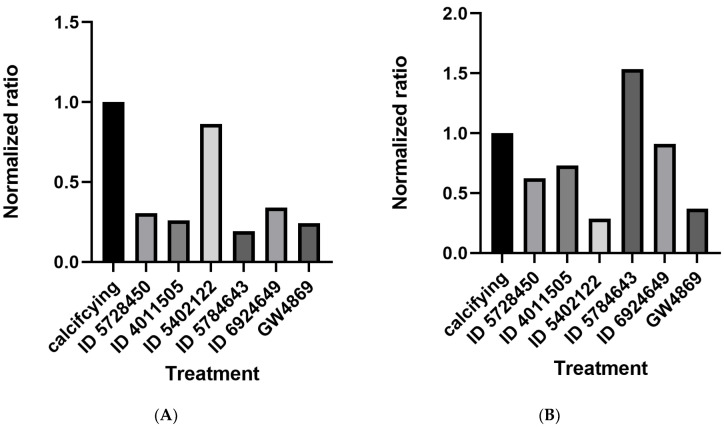

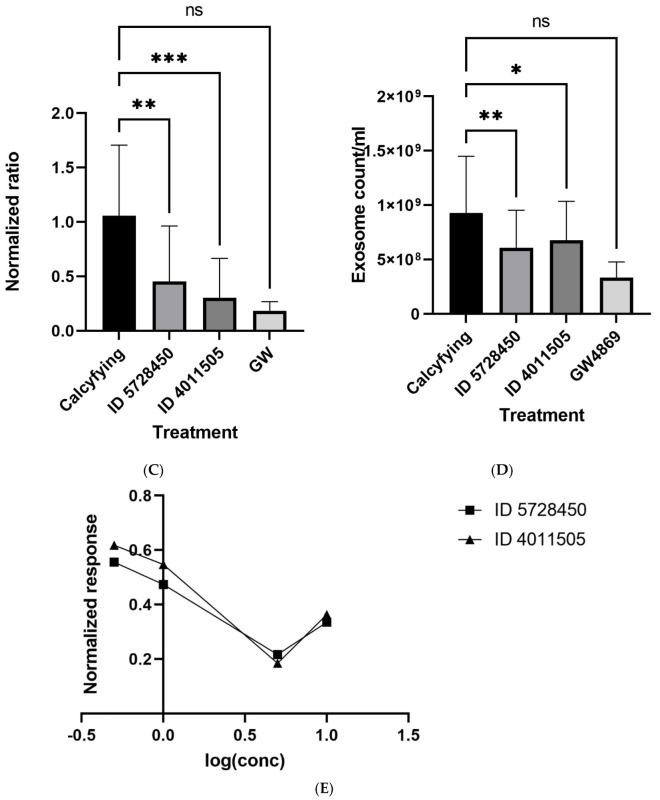
**nSMase2 inhibitors, ID 5728450 and ID 4011505, reduce calcification and EV release in VSMCs.** VSMCs were treated with the test compounds in media with 0.5% FBS and 5.4 mM Ca^2+^ for 48 h. (**A**) Calcification assay with VSMCs treated with 5 µM of each compound. Ca^2+^ concentrations (µg/µL) were normalized to protein concentrations (µg/µL). (**B**) EV quantification using NTA. VSMCs were treated with 5 µM of each compound. (**C**) Calcification assay with VSMCs treated with 5 µM of each compound. Ca^2+^ concentrations (µg/µL) were normalized to protein concentrations (µg/µL); *p* = 0.0060 (ID 572845) and *p* = 0.0005 (ID 4011505) to calcifying control, *p* = 0.0004 between columns. (**D**) EV quantification using NTA. VSMCs were treated with 5 µM of each compound; *p* = 0.0189 (ID 4011505) and (ID 5728450) *p* = 0.0030 to calcifying control, *p* = 0.0073 between columns. (**E**) Calcification assay with VSMCs treated with increasing concentrations of inhibitors. Dose-response calculations indicated IC_50_ = 1.732 µM for ID 5728450, and IC_50_ = 1.910 µM for ID 4011505 with calcification as a readout. Statistical significance was tested using mixed effects analysis (REML) using GraphPad Prism 9.0.2; * *p* < 0.05, ** *p* < 0.01, *** *p* < 0.001.

We next aimed to investigate the activity of the five compounds (two inhibitors, one activator, and two inactive controls) in VSMCs. The effects of compounds on nSMase2-mediated EV release in VSMC was examined, employing an established model that relies on the induction of EV release and calcification by high Ca^2+^ concentrations in culture media [9,11]. The experimental readout was performed through quantitation of EV release and Ca^2+^ deposition in the extracellular matrix. As hypothesized, compound IDs 5728450 and 4011505 both reduced EV release and calcification (Figure 6A,B). The inactive compound ID 5402122 did not change VSMC calcification but, unexpectedly, reduced EV release (Figure 6B). ID 5784643 inhibited calcification, but incubation of VSMC with this compound resulted in enhanced EV release. Finally, compound ID 6924649, which we previously determined to be an activator of nSMAse2, inhibited calcification and showed no effect on EV release from VSMCs.

Both tested inhibitors (ID 5728450 and ID 4011505) were further investigated for their effects on calcification in VSMCs. Both inhibitors significantly reduced calcification at a concentration of 5 µM, with *p* = 0.0060 and *p* = 0.0005, respectively (Figure 6C). Moreover, compound IDs 5728450 and ID 4011505 significantly reduced EV release (*p* = 0.0030 and *p* = 0.0189, respectively; Figure 6D). However, under these conditions, GW4869, the control inhibitor used here, did not show any significant effect on EV release or calcification.

Next, we aimed to determine the specificity of the effects found and performed a dose-response experiment, in which varying concentrations (0.5 µM, 1 µM, 5 µM and 10 µM) of compounds were used to assess their influence on VSMC calcification. This resulted in an IC_50_ of 1.732 µM and 1.910 µM for ID 5728450 and ID 4011505, respectively (Figure 6E). Taken together, these in vitro findings confirmed the inhibitory activities of ID 5728450 and ID 4011505 and validate their use for inhibition of nSMase2.

## 3. Discussion

In this study, we show that human nSMase2 has a druggable pocket. Furthermore, we describe two novel inhibitors of nSMase2, with an IC_50_ in the low micromolar range and druglike properties. We show that these compounds decrease nSMase2 activity in an enzymatic assay and decrease VSMC EV release and calcification in an in vitro model.

### 3.1. Druggable Pocket in nSMase2

This paper describes a combined an in silico-in vitro approach to discover inhibitors of nSMase2 from a large library of small molecules. This method yielded lead compounds that serve as starting points for the development of new drugs, as previously achieved in various drug discovery campaigns [27,28,29,30,31]. The initial step in this process involves structural analysis. In the case of nSMase2, the crystal structure 5UVG was selected as the initial target [26]. At the time this work was carried out, this was the best available experimental structure. To verify the presence of druggable pockets more accurately, 5UVG was refined by a computational approach.

### 3.2. Compounds ID 5728450 and ID 4011505 Are Promising Novel Inhibitors of nSMase2

The procedure described in this paper yielded five compounds that inhibit nSMase2 activity, with IC-50 values in the low µM range, as measured by the enzymatic assay composed of purified reaction components. Although the compounds were selected based on their ability to dock to the active site of nSMase2, we cannot exclude inhibitory mechanisms other than competitive inhibition, and the compounds may bind to other parts of nSMase2. Verification of binding to the intended active site may be experimentally obtained by providing co-crystals of nSMase2 and the selected inhibitors. However, such experiments are beyond the scope of the present work.

The structural features of the discovered inhibitors have been investigated by performing cluster analysis and redocking within the set of compounds that have been experimentally tested in this study. Compound ID 5728450 clustered with 12 other compounds with mostly different activities than itself. Structural characteristics are to be compared among the cluster members, and will be further utilized in separate study for ligand optimization. On the other hand, compound ID 4011505 clustered with only one other compound, which differed structurally only in the position of a methyl group and expressed similar inhibitory activity (normalized activities were 0.152 and 0.060, respectively, as determined by enzymatic assay). This makes the scaffold of ID 4011505 a suitable candidate for further optimizations, to yield even better nSMase2 inhibitors. Such optimizations can be carried out through similar searches of available compound libraries, e.g., from the Zinc Database (containing > 230,000,000 compounds) or, more conventionally, through one or more rounds of targeted synthesis of new compounds following the already established SAR [32].

Redocking of selected compounds showed that, despite the relatively few compounds for which a complete structure-function analysis could be performed, thus preventing the generation of a comprehensive quantitative structure activity relationship (QSAR) analysis, we did observe some trends in the docked poses. The active inhibitors tended to bind to a zone in the active site, which we describe here as zone I. An exception to this was ID 5274372, which appeared to adapt a different orientation in the active site. All poses, however, included a nearness to the active site Mg^2+^ in an area where the nSMase2 substrate SM binds [26]. The two compounds that did not influence the nSMase2 activity appeared to dock to a zone II different from those where the inhibitors or activator bound, whereas the activator bound to a zone III overlapping the zone I but, in addition, appeared to make contact inside a subpocket towards the residue Asn512. Not only topologically, but also in terms of the numbers of contacts made by the compounds, did we observe apparent differences between the three types of compounds tested, with the inhibitors and activators making more contacts to the nSMase active site than the compounds that did not influence enzyme activity. This SAR study illustrates that highly accurate but more rigorous experimental methods, such as co-crystallography of the lead compounds with nSMase2, is needed to obtain the precise binding modes for these compounds. In addition, MD simulation of the experimentally determined cocrystals may be needed, to investigate the dynamic motions and interactions of the lead compounds with nSMase2.

The inhibitors of nSMase2 published thus far display suboptimal properties, such as high IC_50_, lack of specificity, high molecular weight, and mediated inhibition dependent on the presence of other molecules [13,15,17,26,33,34]. The most frequently used pharmacological inhibitor of nSMase2 is GW4869. GW4869 is a non-competitive and non-specific inhibitor with a MW of 577.5 Da and poor aqueous solubility [18], which reduces the phosphatidylserine-induced activation of nSMase2 and nSMase3 [26,33,35]. Its activity is characterized by an IC_50_ of 1 µM; however, due to its lack of specificity, the inability to inhibit nSMase2 directly, and relatively high MW, GW4869 is a suboptimal compound in inhibition of nSMase2-induced signaling pathways. Another known inhibitor is spiroepoxide, an irreversible and non-specific inhibitor (also inhibits Ras farnesyltransferase), with an IC_50_ of 29 µM [13,15,16]. Cambinol has an IC_50_ of 5 µM. It is a more potent nSMase2 inhibitor than manumycin A, which is one of the first discovered nSMase2 inhibitors which is characterized by an IC_50_ of 145 µM [18]. On the other hand, cambinol lacks selectivity, as it also inhibits NAD-dependent deacetylase [34]. A recently published novel compound DPTIP, derived from HTS, has a promising potential to become a druglike candidate, with an IC_50_ of 30 nM; however, the optimization of the compound is still an ongoing process [36]. Thus, ID 5728450 and ID 4011505 identified here have exceptional properties as compared to known nSMase2 inhibitors, due to their specificity, proposedly direct interaction with nSMase2, and low IC_50_.

### 3.3. ID 5728450 and ID 4011505 Inhibit VSMC Calcification and EV Release

Hit compounds identified from the enzymatic assays performed in reaction systems composed of purified components were validated in VSMC-based EV release and calcification assays, confirming the inhibitory effects of compounds ID 5728450 and ID 4011505 in a complex experimental biological setting. Our results are in line with studies showing that inhibition of nSMase2 with GW4869 reduces calcification in vitro [8] and attenuates atherogenesis in vivo [37]. However, GW4869 inhibits nSMase2 indirectly by inhibiting the PS-induced allosteric activation of nSMase2, which results in lower and shorter inhibition times, as well as in a possible interference with pathways upstream of nSMase2. This possibly explains why, in our assays, GW4869 did not inhibit VSMC EV release or calcification, and shows that ID 5728450 and ID 4011505 are better inhibitors. We have not compared their effectiveness in inhibiting EV release and calcification to the other known nSMase2 inhibitors. However, it is known that spiroepoxide can inhibit VSMC calcification in vitro [38].

The VSMC-based assay provided unexpected results with regards to the three additional tested compounds that were selected from clustering analysis. Deemed inactive or an activator, they had disparate effects, especially with respect to EV release. As the EV release and calcification signaling pathways have yet to be fully elucidated, a conclusive remark on the effect of the compounds cannot be made at this stage. Therefore, these compounds should be examined in further cell-based assays, preferably with a knockout of nSMase2, to conclude whether compounds induce cellular effects through directly influencing the nSMAse2 activity. Likewise, further research is needed into the cytotoxicity, stability, and hepatotoxicity of the two discovered hit molecules before they can be described as lead compounds.

## 4. Materials and Methods

### 4.1. Target Selection and Preparation

An optimized crystal structure of the human nSMase2 catalytic domain, in its calcium-bound conformation, was retrieved from the PDB-Redo database (accession code PDBId:5UVG) [39]. This was used as the starting structure for the virtual screening campaign aimed at discovery of small molecule inhibitors of nSMase2. The 5UVG structure does not cover the full length of the catalytic domain, as data on 10 amino acids (positioned at 493–497 and 556–560) out of the total 371 amino acids are lacking [26]. We therefore generated a complete 3D model of the nSMase2 catalytic domain using the YASARA WHATIF Twin package, version 20.10.4 [40,41]. To provide a representative structure for active nSMAse2, we replaced the Ca^2+^ atom from the 5UVG structure by Mg^2+^, which is required for nSMAse2 catalytic activity [42]. Further, the nSMAse2 crystal structure (5UVG.pdb) contained three catalytic water molecules, which were kept in place during molecular dynamics (MD) simulations and docking procedures.

To enable a multiple-conformer conformation virtual screening approach, we employed MD simulation to generate diverse structures of nSMase2. The starting structure was simulated for a total of 100 ns with the AMBER (Assisted Model Building Refinement-force field family) version 16 package [43] Standard parameters (i.e., Amber14ffSB force field, explicit TIP3P waters) and protocols, as comprehensively described in our recent work, were utilized to perform MD simulations [44]. After every 10 ns of simulation time, a structure was saved for pocket inspection by DogSiteScorer to inspect the structure for the presence of potential pockets [22,23].

### 4.2. Ligand Preparation

The Express-Pick ChemBridge compound library, at the start of this project, consisting of 486,844 purchasable compounds and was used to perform a virtual screen for binders to the active site of nSMase2. Prior to actual docking, the library was filtered with FAFDrugs4 [45]. This was done to ensure only small molecules were included that are druglike, as judged by their predicted ADME-Tox (absorption, distribution, metabolism, excretion, and toxicity), pharmacokinetic properties, and Lipinski rule scores, while pan-assay interference compounds (PAINS) were removed [45,46,47]. For each compound in the library, one 3D conformer was generated using Open Babel (version 2.4.0) [48]. The filtered 3D ligands were then optimized for flexible docking, and 10 poses were generated by GLIDE (Schrödinger, version 4.0) for each ligand, following the GLIDE ligand preparation protocol [49]. For all compounds, their poses were flexibly docked on the selected target structures as described below, utilizing three different GLIDE protocols consecutively (see Table 5).

### 4.3. Virtual Ligand Screening

Virtual screening was performed using Schrödinger GLIDE software (Schrödinger, version 4.0) [49]. The docking procedure used a grid with a radius of 8 Å around the catalytic metal ion, and was set up after an initial docking of the natural substrate of nSMase2, sphingomyelin (Figure S1). In docking, we generated 10 ligand poses for each of the eight target protein structures, of which only the complex with the lowest binding energy was used as representative.

In the screening procedure, three docking protocols were successively applied: high throughput virtual screening (HTVS), docking with a more accurate standard precision (SP), and docking with an extra precision protocol (XP) [49]. Each consecutive protocol shows increasing accuracy and specificity, as was determined via extensive sampling, scoring and final elimination of false positives within GLIDE.

For each target protein structure, the filtered compound library was docked by HTVS. After scoring, the highest-ranking 50,000 compounds were docked using the SP protocol. Here, and in the next step, we used four diverse target structures as generated by the MD simulation (i.e., optimized crystal structure and the MD structures after 40 ns, 50 ns, and 80 ns of simulation time, representing the original structure, the structure with the largest pocket volume and structures with high pocket scores). While in the SP and XP steps, higher accuracy in docking was achieved; this came at a cost of increased calculation time, which is why we reduced the number of target structures from eight to four. Finally, the top 5,000 ranked compounds from SP were subjected to the XP step. For the final selection, we visually inspected the 1,500 top scoring compounds. Final compound selection was done by consensus ranking, combining the outcomes of docking to the different target structures and visual inspection of the docked poses [20]. The 996 highest consensus-ranking compounds were purchased from Chembridge Corporation (San Diego, CA, USA) and were used for in vitro screening, using a bioassay for sphingomyelinase activity, as described below.

### 4.4. Redocking and Binding Mode Prediction

**Figure 7:**
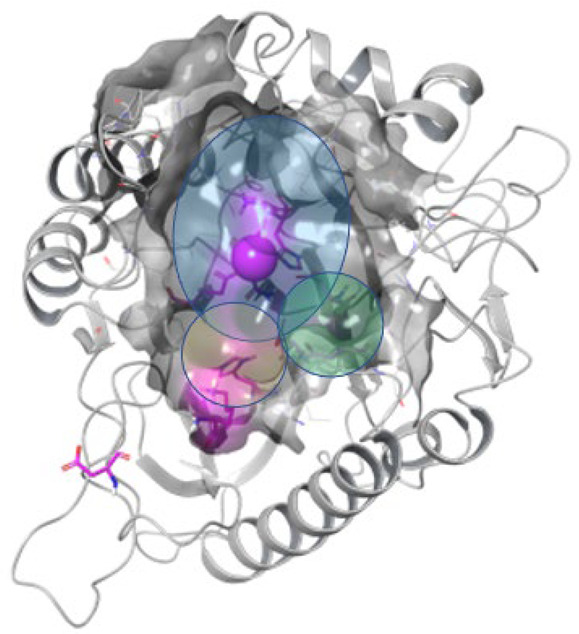
**Illustration of the target pocket to which compounds were redocked.** The target pocket includes the substrate binding pocket that is characterized by its hydrophobic character and the presence of the primary Mg^2+^ ion (Magenta sphere), which is essential for the catalytic activity of nSMase2. The area accessible for small molecules is arbitrarily divided into three zones: I, II and III, illustrated by the blue, yellow and green shading, respectively.

To study the predicted binding modes and interactions of small molecules with nsSMase2 in detail, hit molecules were flexibly redocked using Surflex (as part of the Sybyl-X software package (Certara, version 2.1.1)), which represents a different methodology from that used in the earlier phases of molecule discovery. For compounds containing a stereogenic center, both enantiomers were drawn. Compound 5402122 contains multiple stereogenic centers and appears to contain a sugar group. Therefore, we drew it as naturally occurring D-glucose. We drew both the beta and alpha forms. In analogy to this, for compound 5728450, we chose the same configuration for the stereogenic centers that were present. Prior to docking, the ligands were prepared by addition of Garsteiger–March charges, to generate the preferred tautomer at pH 7 and expanded N and P inversions. We then generated a conformational sample, of which (at most) the five most diverse conformations were kept. As the target structure, we chose the 50 ns MD simulation snapshot, to which Amber 7 FF99 charges, embedded in the Surflex docking software, were added. Additionally, a charge of +2 was added to the Mg atom. A protocol was generated, based on the Mg-ion in the active site. We used a threshold of 0.50 and set bloat to 0. For docking, we used the Surflex-Dock GeomX (SFXC) method [50]. We allowed for movement of hydrogens and heavy atoms. The number of poses to optimize was set to five, and additional starting conformations was set to five as well. We opted for a maximum of 10 poses per ligand, with a minimum RMSD between final poses of at least 0.5. The other parameters were unchanged. The target site includes the active site pocket, as is described in Airola et al. [26], which is formed around the metal ion that is coordinated, in particular, by Asn130 located in the first β-strand of the catalytic domain, and which is essential for catalytic activity. Furthermore, also included is the hydrophobic groove, which binds the substrate SM. Overall, we observe that this groove can be roughly divided into three areas, which we named zone I, II and III, of which zone I and II are relatively shallow (blue and yellow zones) and zone III includes a deeper subpocket pointing towards residue Asn512 at the bottom of this subpocket (Figure 7, Green zone).

### 4.5. In Vitro Compound Testing

All compounds were dissolved in 100% DMSO (Molecular Biology Grade DMSO D8418, Sigma Aldrich, St. Louis, MO, USA) and stored at −20 °C as either 10 mM or 2.5 mM stocks. To reduce the total number of in vitro tests, we prepared 249 mixes of four compounds, at a concentration of 2.5 mM of each compound in the mixture. In a number of compound mixtures, solubility of one or more compounds at room temperature was low; in such cases, the compounds were mixed at a final compound concentration of 20 µM in the mixture in 100% DMSO. Functional activity of the compounds was tested with a commercial Neutral Sphingomyelinase Activity Assay Kit K1800 (Echelon Biosciences, Salt Lake City, UT, USA) according to the manufacturer’s protocol, with minor modification. In short, purified nSMase2 was preincubated with mixes of compounds or individual compounds in the assay buffer at room temperature for 30 min, after which the samples were assayed using standard procedure. The final percentage of DMSO in the assays for all samples and controls tested was 6%. Blank values were obtained by inclusion of buffers with the same percentage of DMSO as in the compound-containing samples. GW4869, a known inhibitor of nSMase activity, at a final concentration of 10 µM, was used as a positive control [35]. To verify if compounds targeted only nSMase2, we included a test sample in which no nSMase2 and no sphingomyelin was present, to observe whether the compounds might interfere with any of the other components of the assay. In this case, 100 µM of phosphorylcholine (PC) was added to allow conversion by alkaline phosphatase (ALP) present in the assay. The formation of a blue reaction product by the assay was spectrophotometrically detected by measuring light absorbance at 595 nm on a microtiterplate reader (Cytation3, BioTek, Santa Clara, CA, USA).

### 4.6. Cell Culture and Calcification Assays

Human primary VSMCs were derived from tissue explants and cultured, as described previously [51], in M199 with 20% FBS and 100 U/mL penicillin, 100 μg/mL streptomycin (Gibco, Invitrogen, Breda, The Netherlands). Human aortic samples were obtained from patients undergoing open aortic surgery at the Maastricht University Medical Centre. Collection, storage, and use of tissue and patient data were performed in agreement with the Dutch Code for Proper Secondary Use of Human Tissue (http://www.fmwv.nl, access date 1 January 2019). This study complies with the Declaration of Helsinki. VSMCs were passaged when 80% confluent and used for experiments at passages 5–12. Medium was changed every 2–3 days. For calcification assays, VSMCs were seeded at 10,000 cells/cm^2^ and conditioned in M199 media-supplemented CaCl_2_ to reach a final concentration of 5.4 mM CaCl_2_ in the presence of 0.5% FBS in M199 media, to accelerate the process of calcification. Compounds were added to cells, along with calcifying media at 0.5, 1, 5, and 10 µM; vehicle controls (DMSO) were also included.

Calcification was measured as previously described [52]. In short, cells were washed twice with PBS, after which the mineralized matrix was dissolved in 0.1M HCl for 2 h. To quantify the amount of Ca^2+^ per sample, the Calcium assay kit (Randox, #CA8309) with o-cresolphthalein was used according to manufacturer’s instructions. Ca^2+^ content in each sample was normalized to protein concentration. All samples were assayed in triplicate in three independent experiments, using a Cytation3 plate reader (BioTek, Santa Clara, CA, USA).

### 4.7. Protein Quantification

Protein concentrations were measured using the DC protein assay (#5000111, BioRad, Hercules, Ca, USA,) according to the manufacturer’s protocol. Samples were prepared by adding 0.1 M NaOH/0.2% SDS (Sigma, St. Louis, MO, USA, #06203; Biorad, Hercules, CA, USA, #1610416) to cell samples suspended in 0.1 M HCl for 2 h. Absorbance was read at 750 nm using Cytation3 (BioTek, Santa Clara, CA, USA). A standard curve was created using BSA. All samples were assayed in triplicate in three independent experiments.

### 4.8. Extracellular Vesicle Quantification

EVs were quantified in media collected from calcification assays using previously established protocols [53]. Briefly, media was aspirated from VSMCs and spun at 500× *g* for 5 min to eliminate cell debris and snap-frozen until further analysis. Further, calibration beads and 50 uL of the supernatants were diluted in PBS to a final volume of 1 mL. EVs quantification was performed using nanoparticle tracking analysis (NTA, ZetaView, Particle Metrix, Inning am Ammersee, Germany). Optimum scanning conditions were established using previously described protocols [53]. A washing step with distilled water was performed between each technical sample reading. Data analysis was performed with NTA 2.1 software (ZetaView, Particle Metrix, Inning am Ammersee, Germany).

## 5. Conclusions

Taken together, we describe here two novel druglike molecules, compounds ID 5728450 and ID 4011505, that express properties outperforming existing nSMAse2 inhibitors, which inhibit VSMC calcification and EV release. The new molecules possess novel scaffold structures that may evolve into new classes of nSMase2 inhibitors. Their discovery represents an essential first step towards a clinical stage of development. Given the implication of nSMAse2 in a vascular calcification, this new class of nSMAse2 inhibitors could contribute to the management of this disease and others mediated by EV release and calcification.

## Figures and Tables

**Table 1:** **Inspection of the binding site volume for 10 MD-derived protein structures in DoGSiteScorer.** The calculation was performed with a grid-based method applying a Difference of Gaussian filter to detect the pockets. This incorporates size and shape descriptors, element descriptors, functional groups descriptors, amino acids composition, and amino acid descriptors [22,23].

Structure Name	Pocket Volume (Å³)	Drug Score
10 ns	835.01	0.81
20 ns	960.9	0.82
30 ns	624.26	0.85
40 ns	630.85	0.84
50 ns	708.22	0.86
60 ns	623.3	0.84
70 ns	962.75	0.81
80 ns	1074.43	0.81
90 ns	724.35	0.74
100 ns	619.78	0.71

**Table 2:** Top five compounds, as identified by their compound ID, their structural formula, and the IC_50_ (in µM), as determined from the nSMase2 enzymatic assay.

Compound ID	Structure	IC_50_ (µM)	SD
5728450	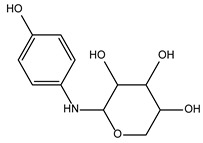	1.841	0.34
5122895	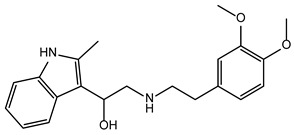	1.586	0.26
5247372	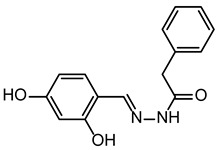	1.744	0.28
5150856	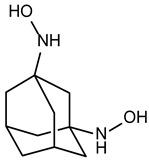	11.74	0.21
4011505	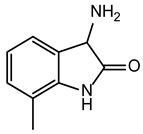	1.001	0.27

**Table 3:** Representative variability of compound structures, as obtained by cluster analysis using Data Warrior [25]. Activity values calculated from experiments in Echelon enzymatic assay.

Compound ID	2D Structure	Normalized Activity	Cluster Number	Property
4011505	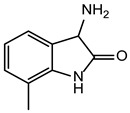	0.152	8	Inhibitor
5728450	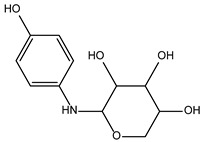	0.121	103	Inhibitor
5402122	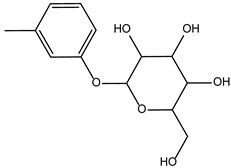	1.007	103	Inactive. Structurally similar to 5728450.
5784643	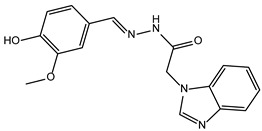	0.885	154	Inactive. Structurally different from 5402122.
6924649	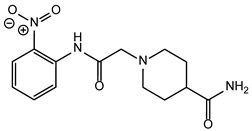	1.461	-	Activator. Did not cluster.

**Table 4:** **Overview and comparison of contacts made between small compounds and nSMase2.** Indicated are compounds’ IDs. The residues in nSMase2 that are involved in interaction with the compounds are shown as shaded amino acid abbreviations. Further, the number of observed hydrogen bonds, salt bridges, metallic interactions, pi-pi interactions, or cation-pi interactions between compounds and nSMase2 are shown, as well as the total number of interactions per compound. For comparison in the bottom line, the residues Asn130, Glu364, Asp510, Asn512, Asp638 and His639 representing conserved active site residues contacting the SM substrate [26] are indicated.

ID	Interacting Residues	Hydrogen Bonds	Salt Bridges	Metallic Interactions	π-π Interactions	Cation-π	Total	Type
5728450	NRNREFKLQDNTRDHMg	5	0	0	0	0	5	inhibitor
5122895	NRNREFKLQDNTRDHMg	3	1	1	0	0	5	inhibitor
5275372	NRNREFKLQDNTRDHMg	3	0	0	0	2	5	inhibitor
5150856	NRNREFKLQDNTRDHMg	4	1	0	0	0	5	inhibitor
4011505	NRNREFKLQDNTRDHMg	3	1	0	0	0	4	inhibitor
5402122	NRNREFKLQDNTRDHMg	3	0	1	0	0	4	neutral
5784643	NRNREFKLQDNTRDHMg	2	0	1	1	0	4	neutral
6924649	NRNREFKLQDNTRDHMg	4	3	0	0	0	7	activator
	**N**RNR**E**FKLQ**DN**TR**DH**Mg							

With NRNREFKLQDNTRDHMg referring to: Asn130, Arg140, Asn143, Arg150, Glu364, Phe366, Lys435, Leu462, Gln466, Asp510, Asn512, Thr554, Arg604, Asp638, His639,catalytic Mg2+ ion.

**Table 5:** Virtual screening strategy.

Number of Diverse Protein Target Structures.	Virtual Screening Protocol	Number of Compounds Docked per Structure
8	High throughput virtual screening	480,000
4	Standard precision	50,000
4	Extra precision	5000

## Data Availability

The data collected in this study are available from the corresponding author upon request.

## Supplementary Materials

The following supporting information can be downloaded at: https://www.mdpi.com/article/10.3390/ijms24032027/s1. Figure S1. Binding pose of sphingomyelin docked in the active site of the model of human nSMase2. (A) 2D and (B) 3D visualization. Figure S2A Medicinal Chemistry_SwissADME for the compound ID 5728450. Figure S2B Medicinal Chemistry_SwissADME for the compound ID 4011505. Figure S2C Medicinal Chemistry_SwissADME for the compound ID 5122895. Figure S2D Medicinal Chemistry_SwissADME for the compound ID 5247372. Figure S2E Medicinal Chemistry_SwissADME for the compound ID 5150856. Figure S3A Graphical presentation of compound clustering in DataWarrior. Figure S3B Graphical presentation for cluster 8 in DataWarrior. Figure S3C Graphical presentation for cluster 103 in DataWarrior. Figure S4A 3D docking pose of compound 5728450 after redocking. Figure S4B 3D docking pose of compound 5122895 after redocking. Figure S4C 3D docking pose of compound 5274372 after redocking. Figure S4D 3D docking pose of compound 5150856 after redocking. Figure S4E 3D docking pose of compound 4011505 after redocking. Figure S4F 3D docking pose of compound 5402122 after redocking. Figure S4G 3D docking pose of compound 5784643 after redocking. Figure S4H 3D docking pose of compound 6924649 after redocking.
