# Supplementary figures and images for: Inhibition of Neutral Sphingomyelinase 2 by Novel Small Molecule Inhibitors Results in Decreased Release of Extracellular Vesicles by Vascular Smooth Muscle Cells and Attenuated Calcification
Source: Int J Mol Sci. 2023 Jan 19;24(3):2027. doi: 10.3390/ijms24032027 (PMC9916533; doi:10.3390/ijms24032027)

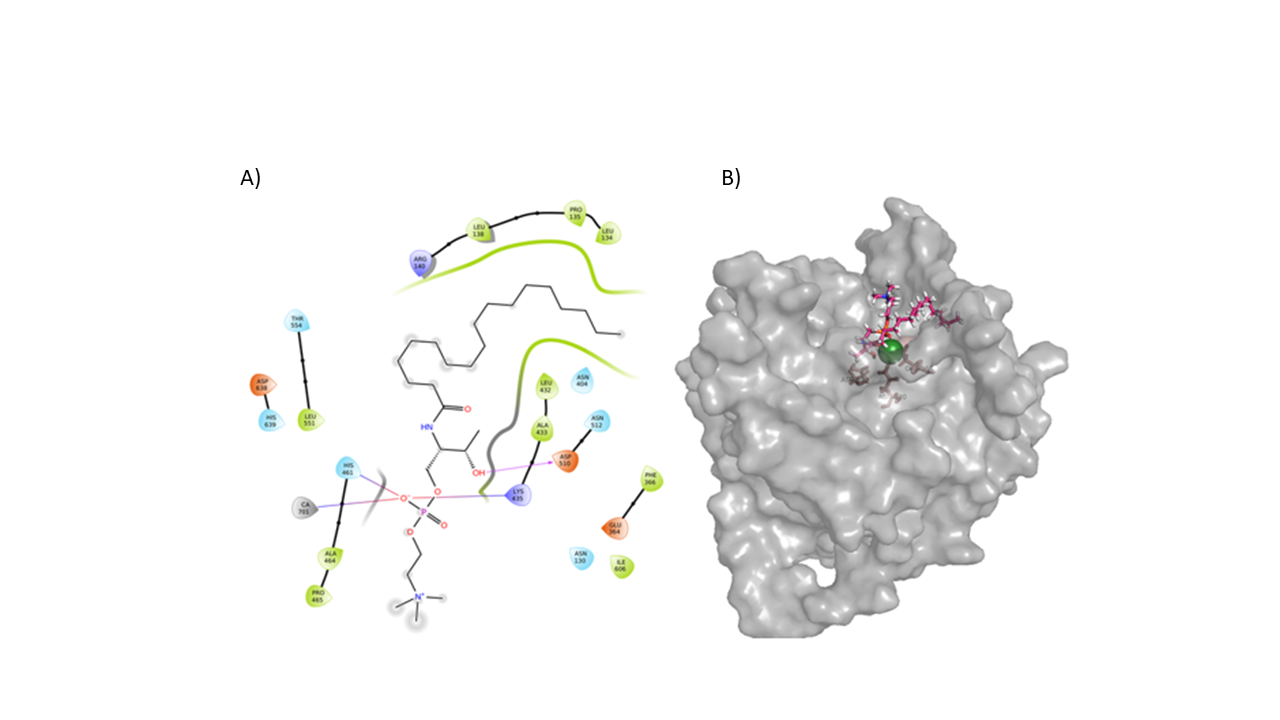

Supplement: Supplementary file 1 [file ijms-24-02027-s001.zip › final supplemental figures/Suppl_fig1AB.tif]

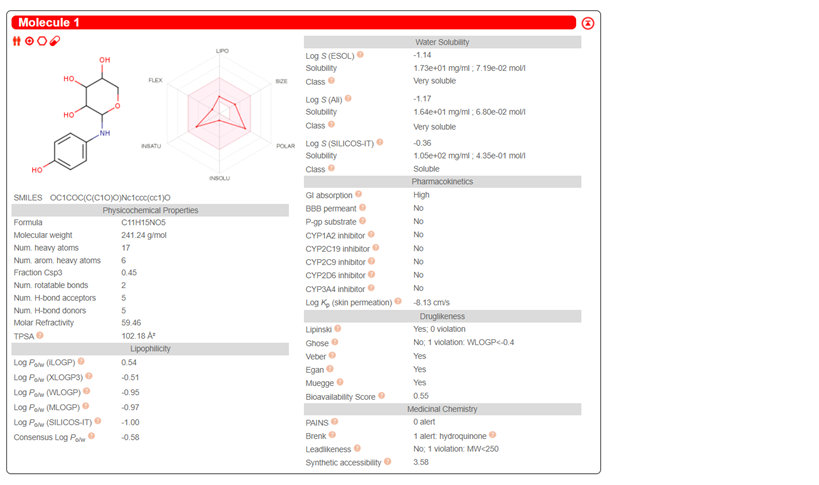

Supplement: Supplementary file 1 [file ijms-24-02027-s001.zip › final supplemental figures/Suppl_fig2A.tif]

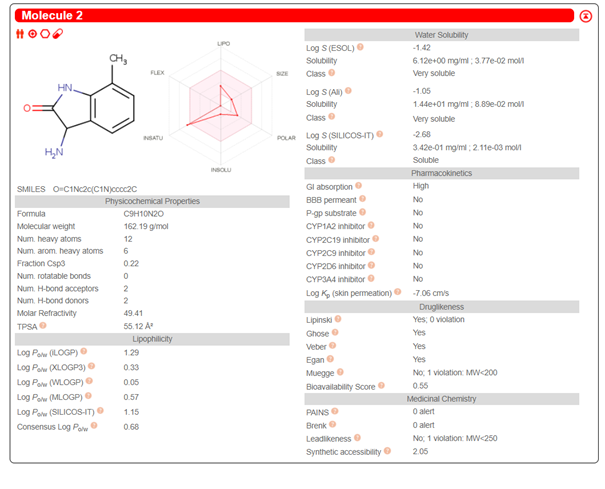

Supplement: Supplementary file 1 [file ijms-24-02027-s001.zip › final supplemental figures/Suppl_fig2B.tif]

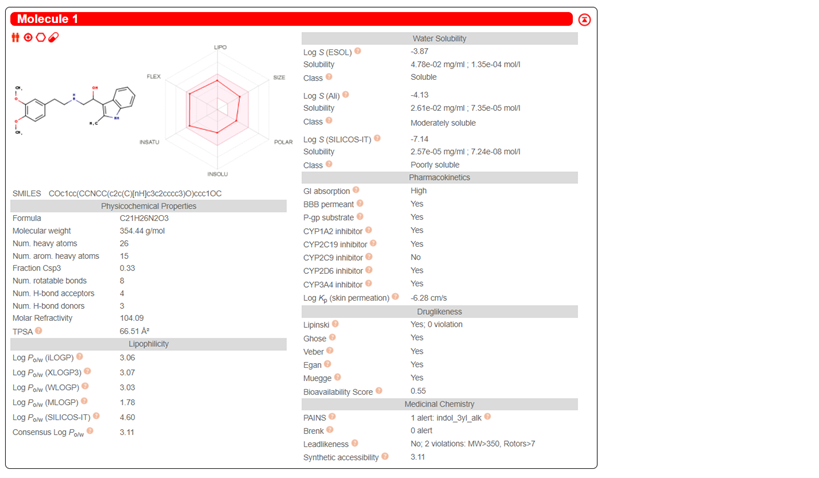

Supplement: Supplementary file 1 [file ijms-24-02027-s001.zip › final supplemental figures/Suppl_fig2C.tif]

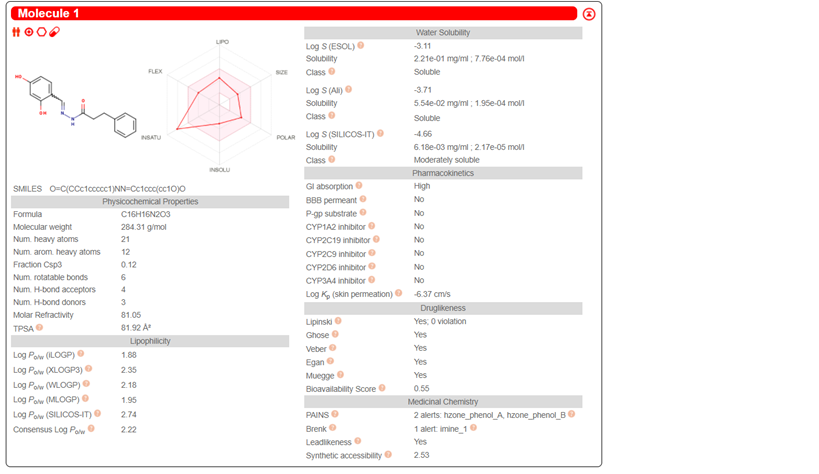

Supplement: Supplementary file 1 [file ijms-24-02027-s001.zip › final supplemental figures/Suppl_fig2D.tif]

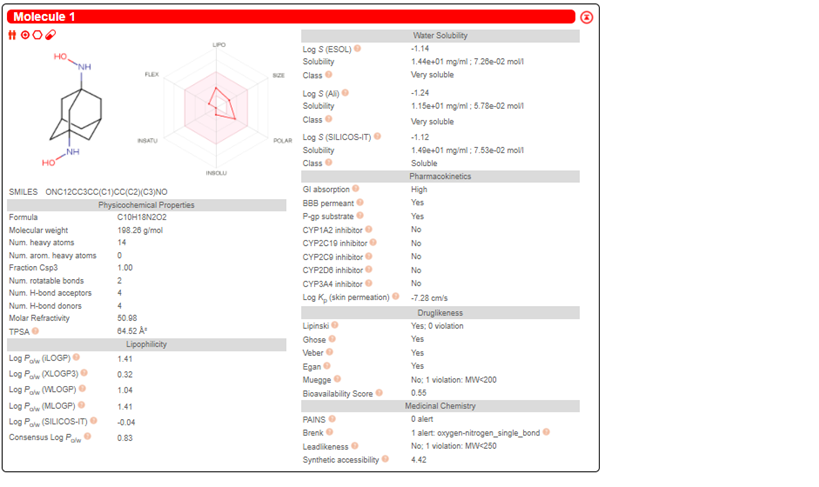

Supplement: Supplementary file 1 [file ijms-24-02027-s001.zip › final supplemental figures/Suppl_fig2E.tif]

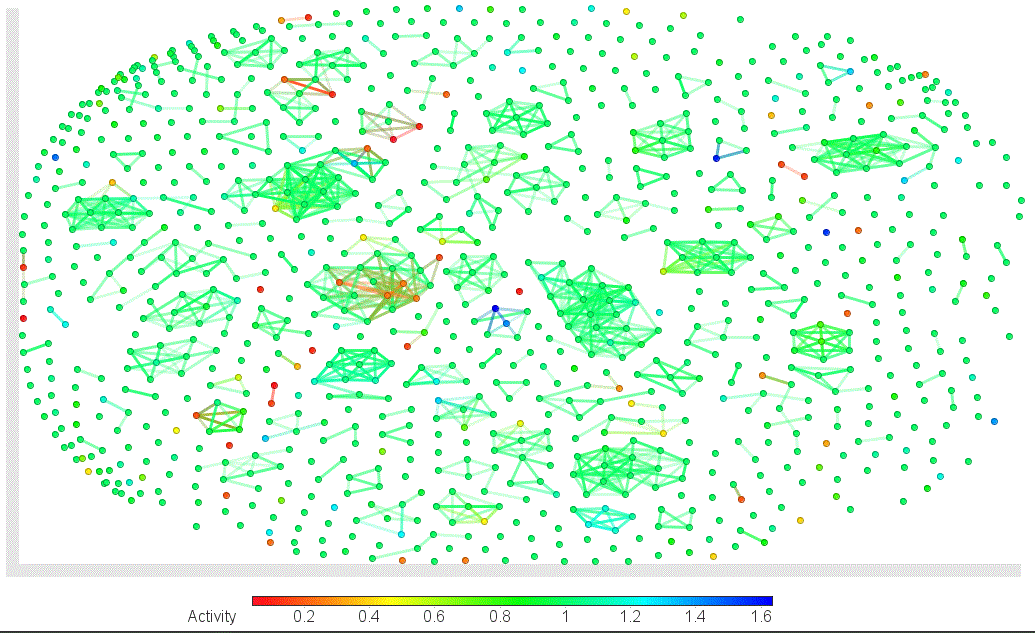

Supplement: Supplementary file 1 [file ijms-24-02027-s001.zip › final supplemental figures/Suppl_fig3A.tif]

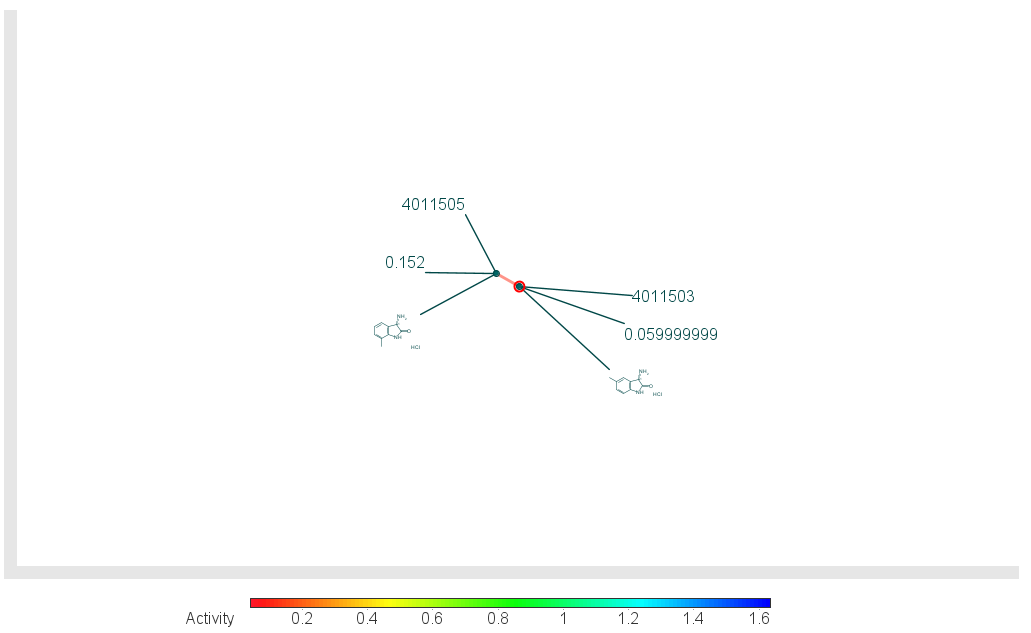

Supplement: Supplementary file 1 [file ijms-24-02027-s001.zip › final supplemental figures/Suppl_fig3B.tif]

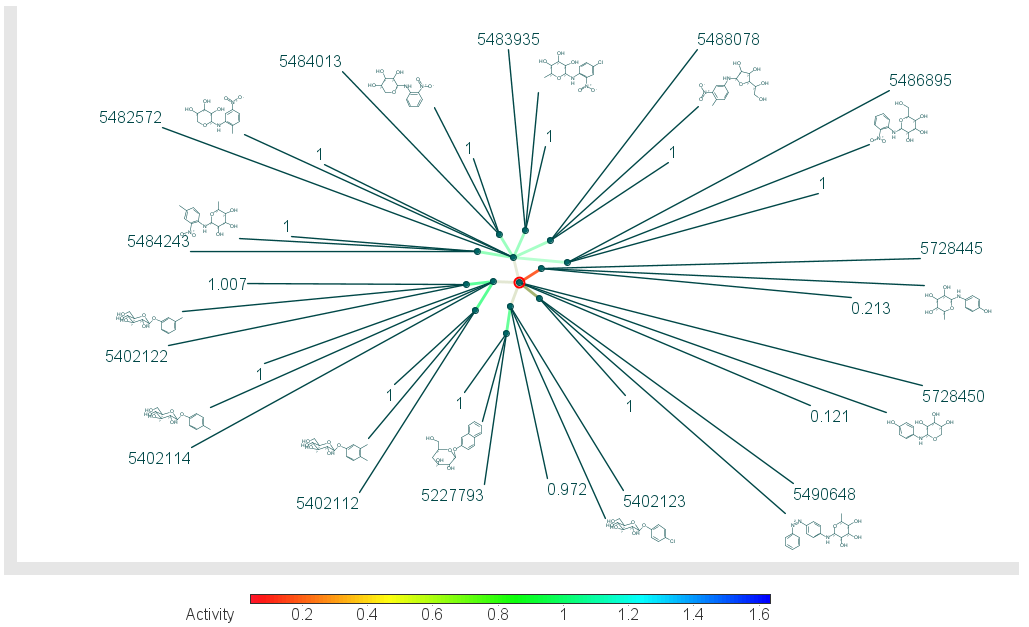

Supplement: Supplementary file 1 [file ijms-24-02027-s001.zip › final supplemental figures/Suppl_fig3C.tif]

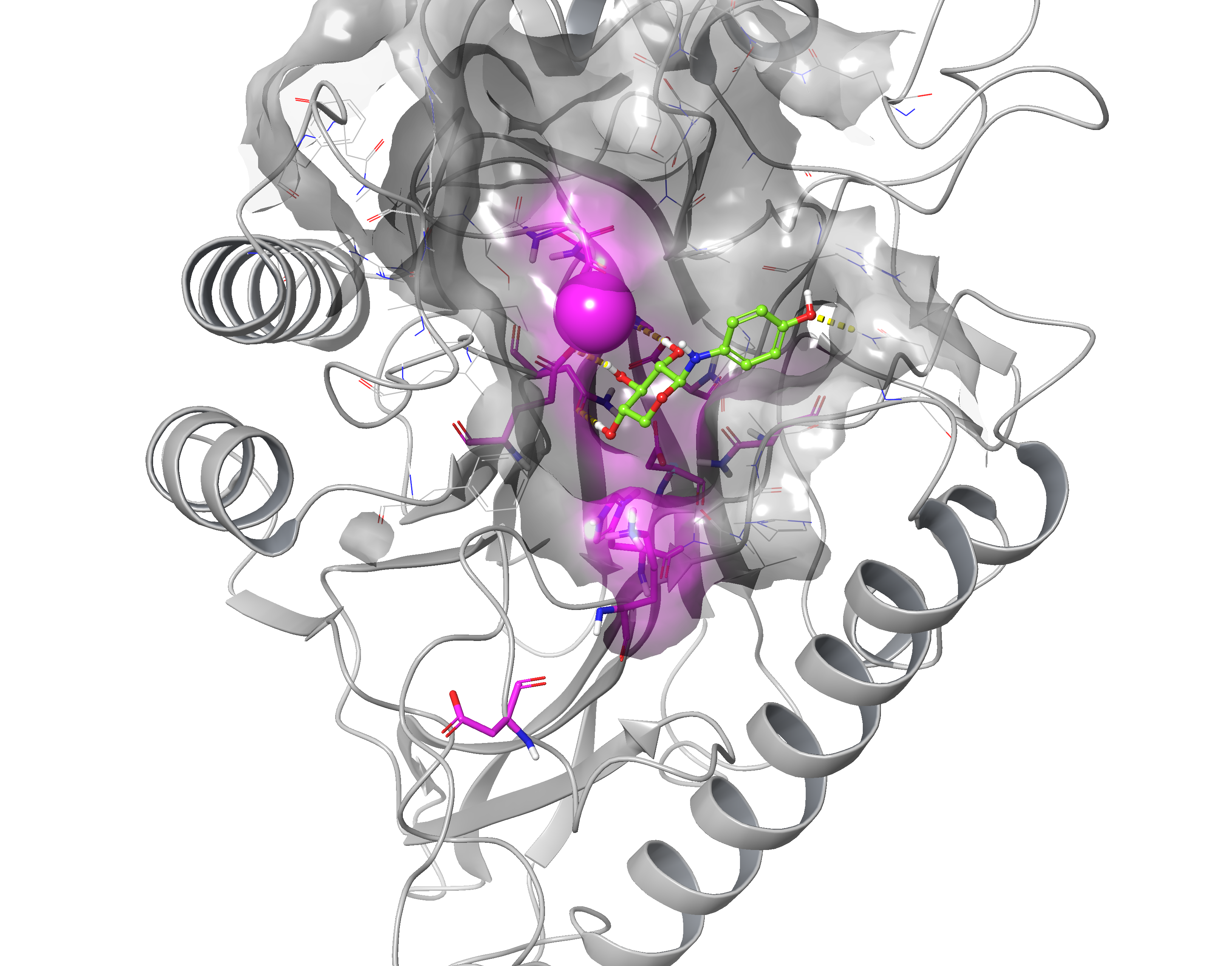

Supplement: Supplementary file 1 [file ijms-24-02027-s001.zip › final supplemental figures/Suppl_fig4A.tif]

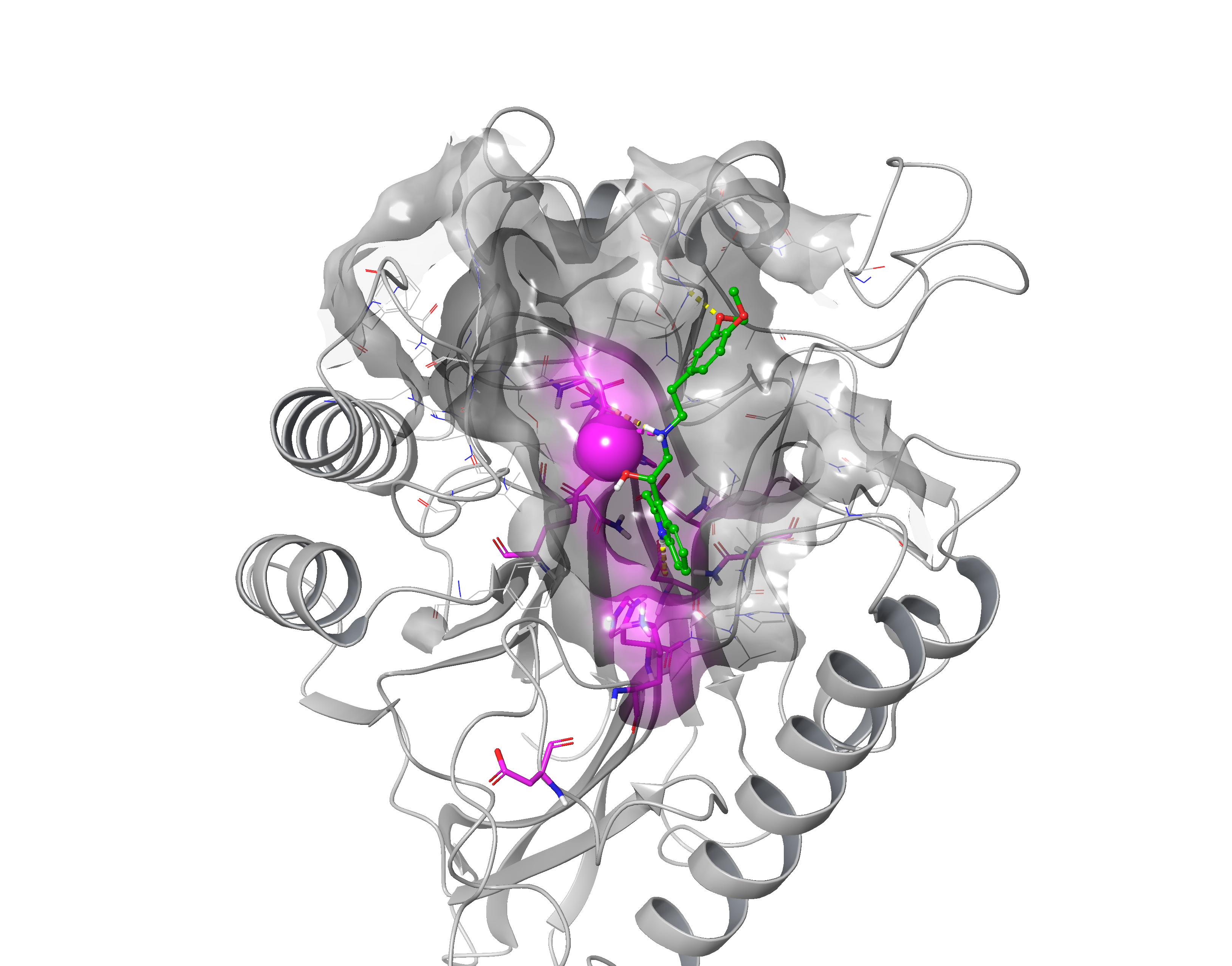

Supplement: Supplementary file 1 [file ijms-24-02027-s001.zip › final supplemental figures/Suppl_fig4B.tif]

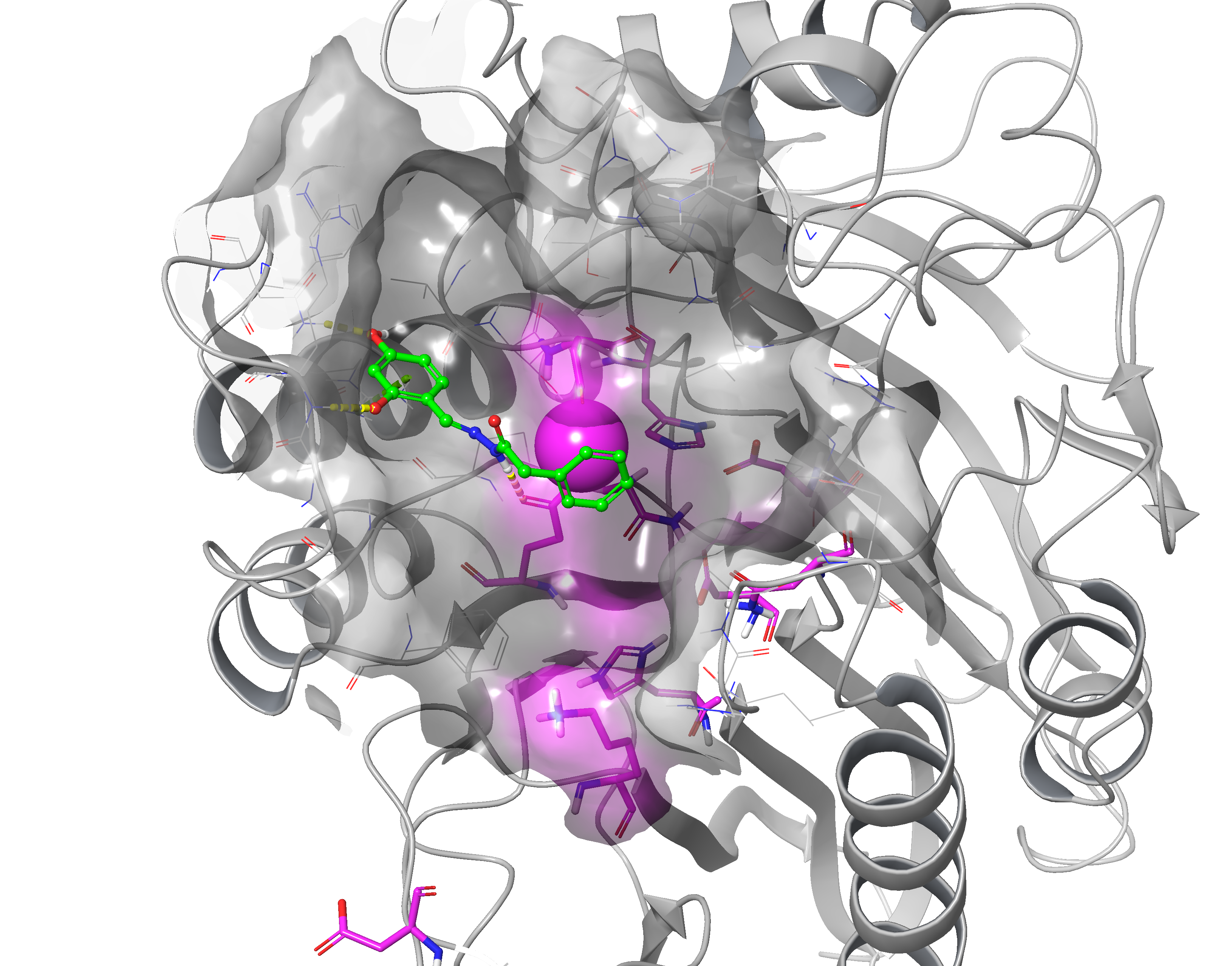

Supplement: Supplementary file 1 [file ijms-24-02027-s001.zip › final supplemental figures/Suppl_fig4C.tif]

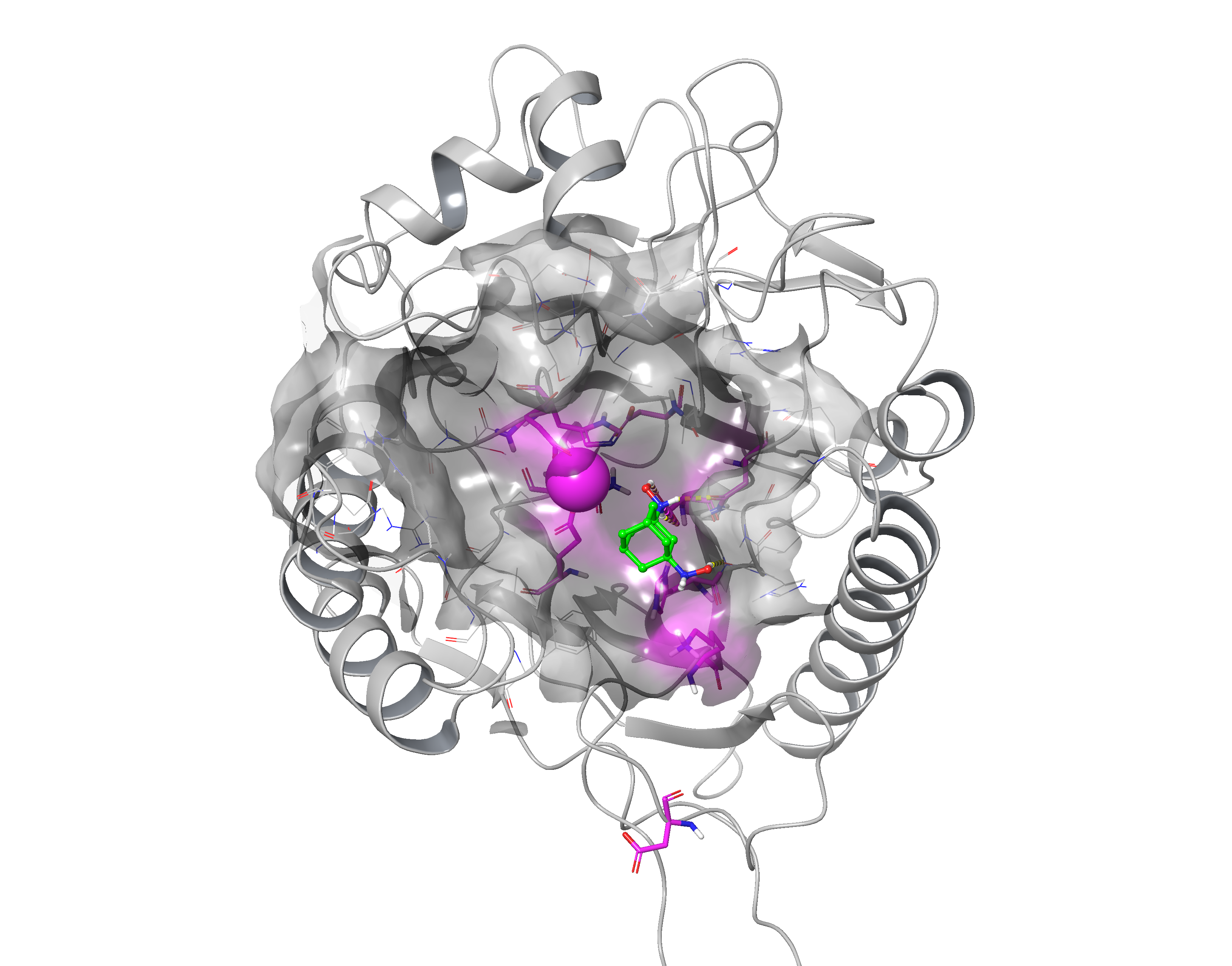

Supplement: Supplementary file 1 [file ijms-24-02027-s001.zip › final supplemental figures/Suppl_fig4D.tif]

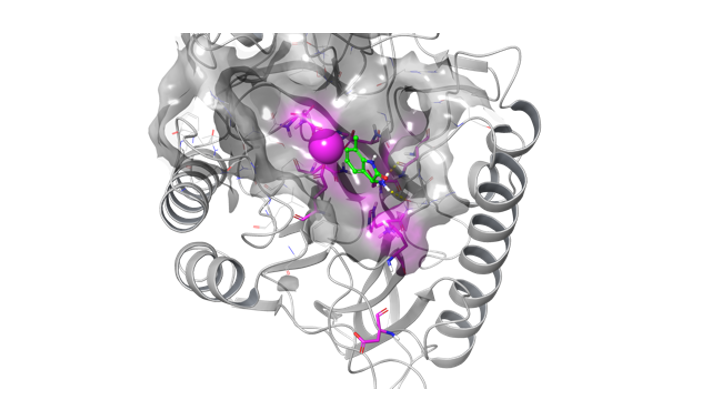

Supplement: Supplementary file 1 [file ijms-24-02027-s001.zip › final supplemental figures/Suppl_fig4E.tiff]

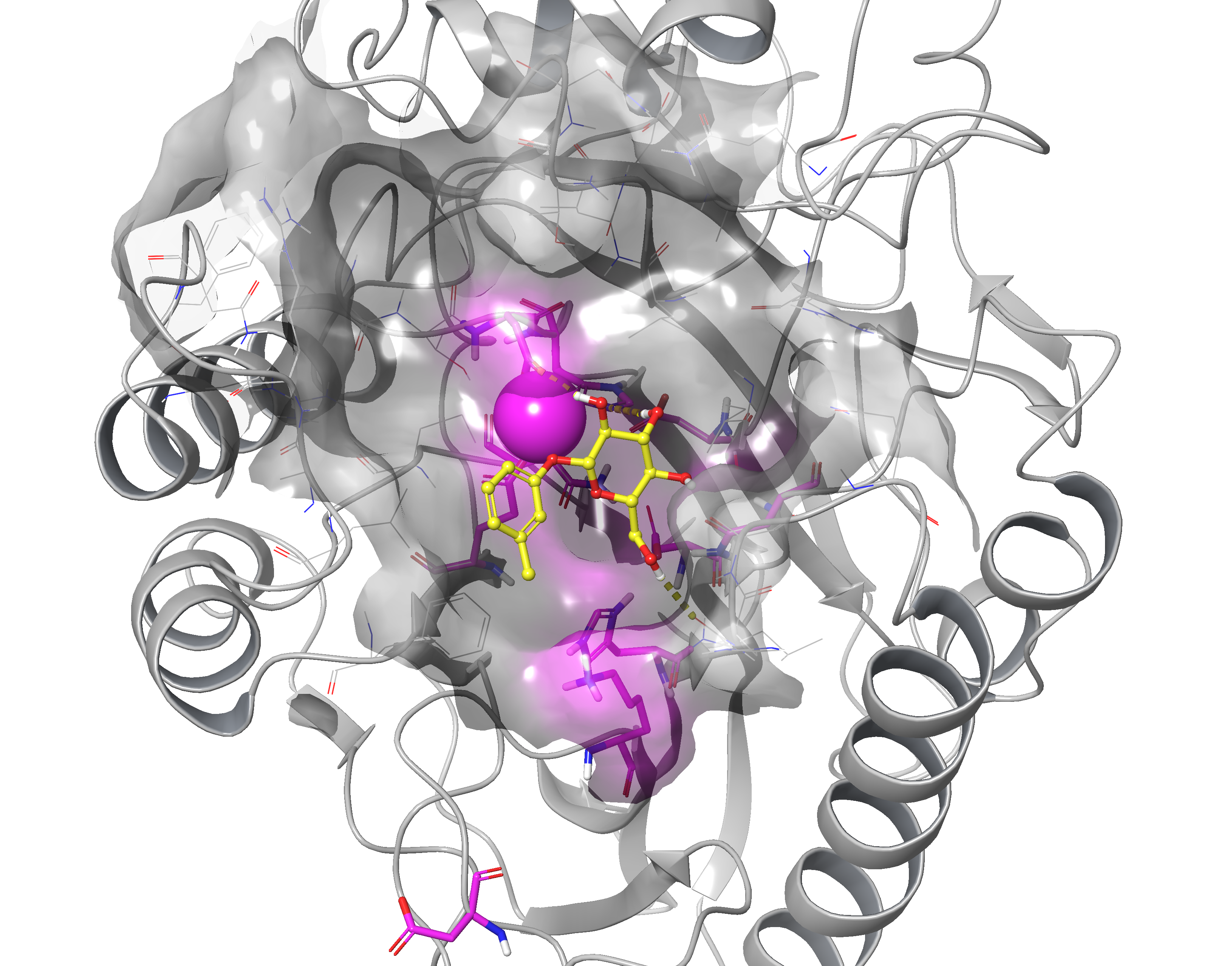

Supplement: Supplementary file 1 [file ijms-24-02027-s001.zip › final supplemental figures/Suppl_fig4F.tif]

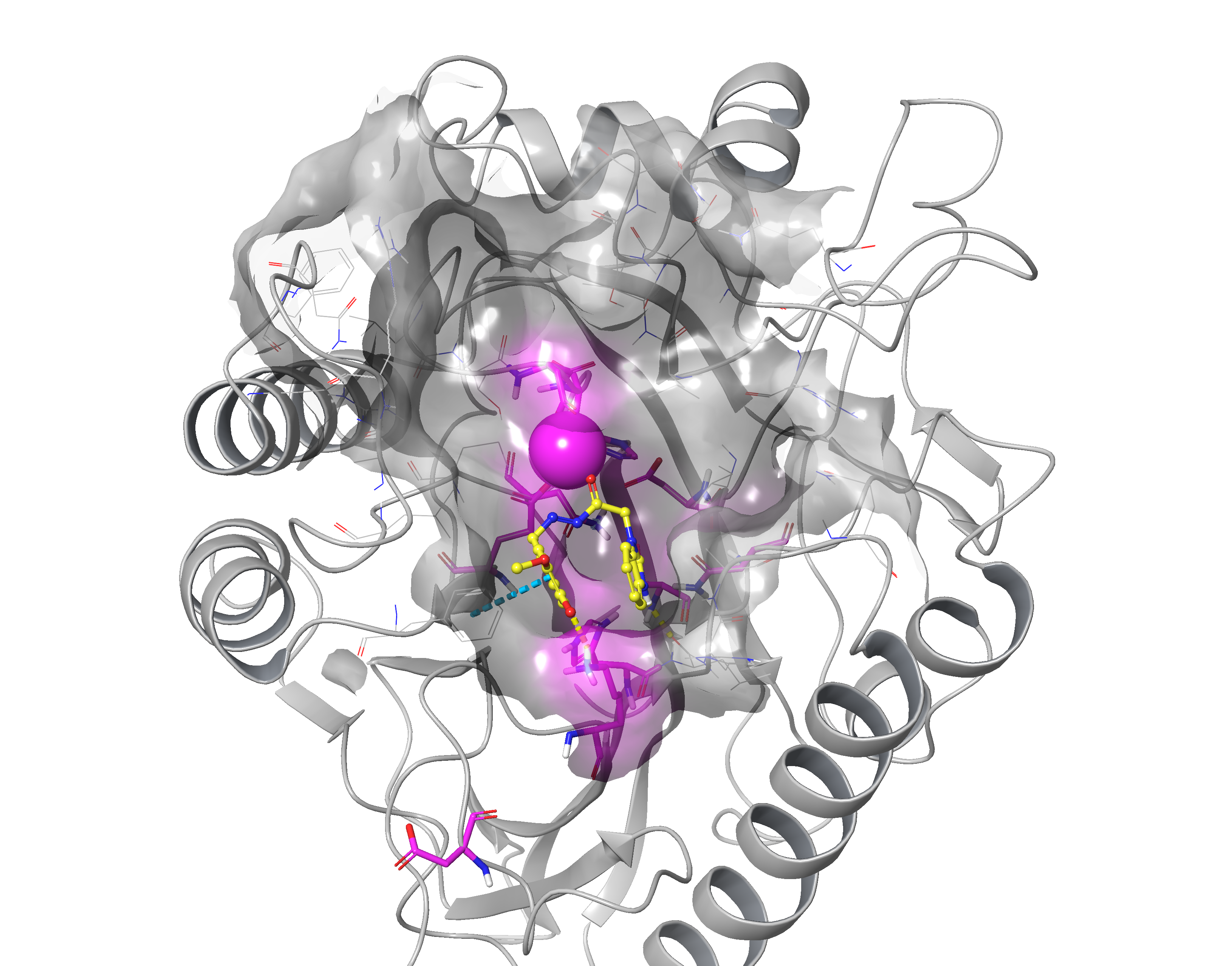

Supplement: Supplementary file 1 [file ijms-24-02027-s001.zip › final supplemental figures/Suppl_fig4G.tif]

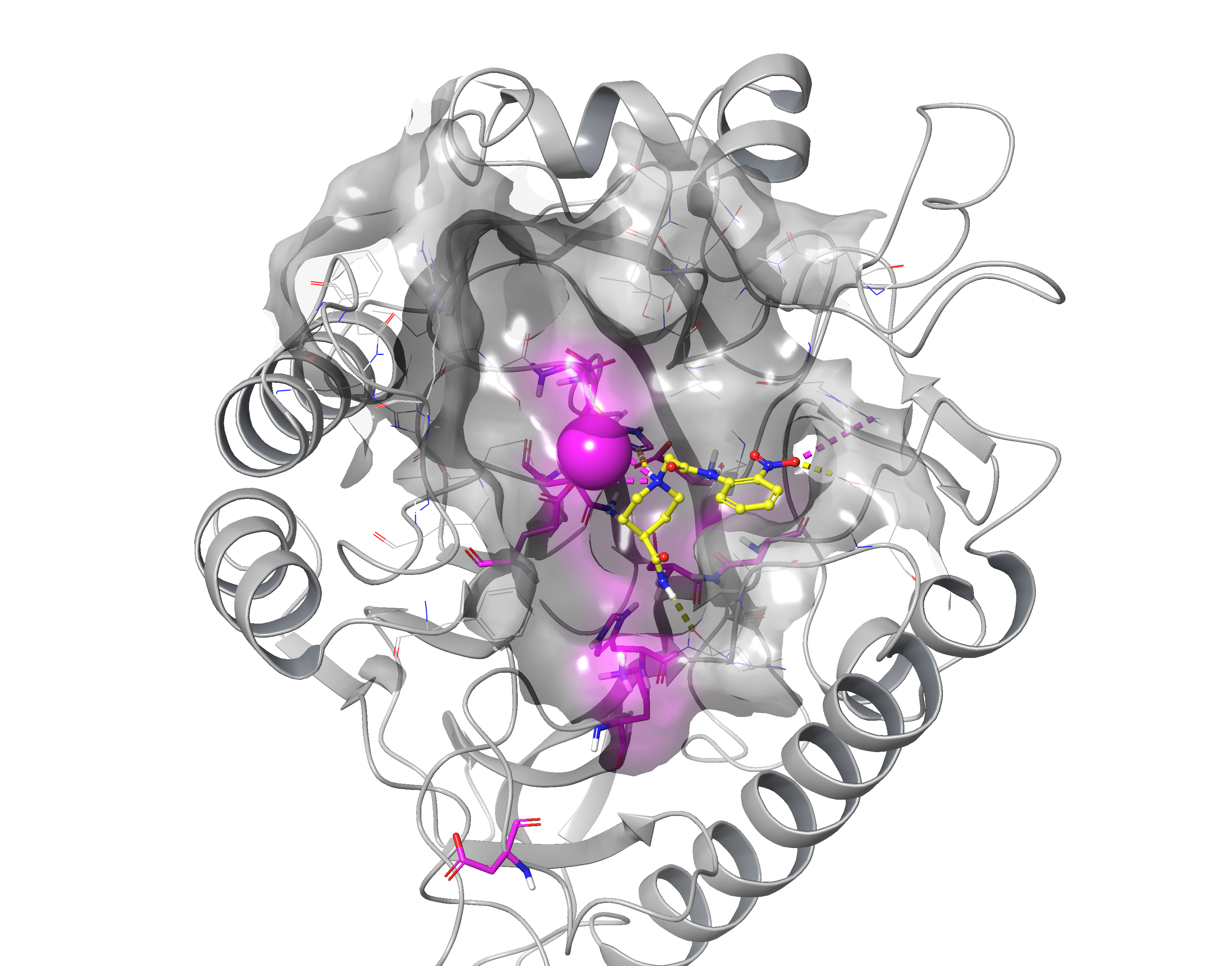

Supplement: Supplementary file 1 [file ijms-24-02027-s001.zip › final supplemental figures/Suppl_fig4H.tif]
